# Molecular link between glucose and glutamine consumption in cancer cells mediated by CtBP and SIRT4

**DOI:** 10.1038/s41389-018-0036-8

**Published:** 2018-03-13

**Authors:** Li Wang, Jing-jing Li, Li-yu Guo, Peipei Li, Zhiqiang Zhao, Haisheng Zhou, Li-jun Di

**Affiliations:** 1 Faculty of Health Sciences, University of Macau, Macau, People’s Republic of China; 2 Department of Biochemistry and Molecular Biology, Anhui Medical University, Hefei, 230032 China

## Abstract

Glucose and Glutamine are two essential ingredients for cell growth. However, it remains open for investigation whether there is a general mechanism that coordinates the consumption of glucose and glutamine in cancer cells. Glutamine is mainly metabolized through the glutaminolysis pathway and our previous report indicated that CtBP increases GDH activity and promotes glutaminolysis through repressing the expression of *SIRT4*, a well-known mitochondrion-located factor that inhibits glutaminolysis pathway. CtBP is known to be a sensor of intracellular metabolic status; we thus hypothesized that a consensus CtBP-SIRT4-GDH axis may mediate the crosstalk between glycolysis and glutaminolysis. Herein, supporting this hypothesis, we observed the coordinated consumption of glucose and glutamine across different cell lines. This coordination was found to be related to CtBP repression activity on SIRT4 expression under high level of glucose but not low glucose level. Low level of glucose supply was found to decrease GDH activity via blocking CtBP dimerization. Mechanically, low glucose also abolished CtBP binding to *SIRT4* promoter and the repression of *SIRT4* expression. Consistently, the CtBP dimerization inhibitor MTOB mimicked low glucose effects on *SIRT4* expression, and GDH activity suggest that CtBP requires high glucose supply to act as a suppressor of *SIRT4* gene. In conclusion, we propose that a general molecular pathway composed by CtBP-SIRT4-GDH coordinating the metabolism of glucose and glutamine in cancer cells.

## Introduction

Glucose and glutamine are critical nutrients indispensable for cancer cell growth^1^. Current knowledge about the metabolism of these two nutrients suggests that they are consumed by the cells through distinct pathways^2,3^. Glucose is transported into cells and further metabolized to pyruvate through the glycolysis pathway. The pyruvate either enters the mitochondria for tricarboxylic acid (TCA) cycle, or it will be converted to lactate. The latter pathway also represents a major advantage for cancer cell growth^4^, even though some other branching pathways such as one carbon metabolism and pentose phosphate pathway (PPP) were also found to be important channels to convert glucose to other essential downstream molecules for cancer cell growth^5,6^. Glutamine is mainly utilized through the glutaminolysis pathway and the research about this pathway has attracted great attention in recent years, because cancer cells were found to rely on this pathway for durable supply of carbon and nitrogen^7^.

The crosstalk between glycolysis and glutaminolysis has been noticed a long time ago; however, how these two processes influence each other is controversial. Previous studies indicated that the interactive activity of these two pathways is mediated by some intermediate metabolites such as pyruvate. Pyruvate is the end product of glycolysis and glutamine can also be used to produce pyruvate; however, the latter process is more complicated and needs to go through several enzymatic reactions belonging to the TCA cycle^8^. The other way of interaction is through serine synthesis pathway^6^. Glutamate provides an amine group to 3-phosphopyruvate, a product converted from glycolysis intermediate 3-phosphoglycerate, to form 3-phosphoserine, the precursor of serine. The third interactive mechanism between glycolysis and glutaminolysis correlates with synthesis of nucleotide hexosamine, a substrate for protein glycosylation, which requires the input from both glucose and glutamine. Wellen et al.^9^ found that glycolysis is required for glutamine uptake in multiple types of mammalian cells and the mediating factor, IL3Ra, was found to be glycosylated by hexosamine that is synthesized dependent on glucose. Consistently, the conclusion of glycolysis promoting glutamine uptake has also been demonstrated in another independent study using B cell as a model. In this study, withdrawal of glucose led to almost 10 times decrease in glutamine metabolism, but the mechanism has not been elaborated^10^.

Recent studies indicate that fast proliferating cells, in particular, the cancer cells, require the durable supply of both energy and metabolites used as “building blocks.” Both glucose and glutamine are consumed to fulfill this requirement^11,12^. Glucose, for instance, goes through the “aerobic glycolysis” process to accelerate the output of ATP. However, glutamine is directly transported into the TCA cycle to exaggerate the output of intermediate metabolites, especially the citrate, which can be further converted to acetyl-CoA as a building block for the synthesis of fatty acid etc. Glutaminolysis mainly occurs in mitochondria where glutamine is converted to α-ketoglutarate and enters the TCA cycle. Upon DNA damage, glutaminolysis is halted temporarily to contribute to cell cycle inhibition^13^. One of the known mediators of DNA damage response in controlling glutaminolysis is SIRT4. SIRT4 is a mitochondrion-localized Sirtuin family protein with both deacetylation and ADP-ribosylation enzymatic activities^14^. SIRT4 catalyzes the ADP-ribosylation of gutamate dehydrogenase (GDH), an enzyme converting glutamate to α-ketoglutarate, leading to repressed glutaminolysis^14^.

Our previous data indicated that *SIRT4* expression is repressed by a transcriptional co-repressor CtBP to contribute to the maintenance of pH homeostasis of breast cancer cells, which benefits cancer cells for their growth^15^. Here we further report that CtBP repression of *SIRT4* expression is regulated by glycolysis activity in cancer cells and highlight a novel pathway that mediates the crosstalk between glycolysis and glutaminolysis.

## Results

### Correlated glucose and glutamine consumption in cancer cells

**Fig. 1:**
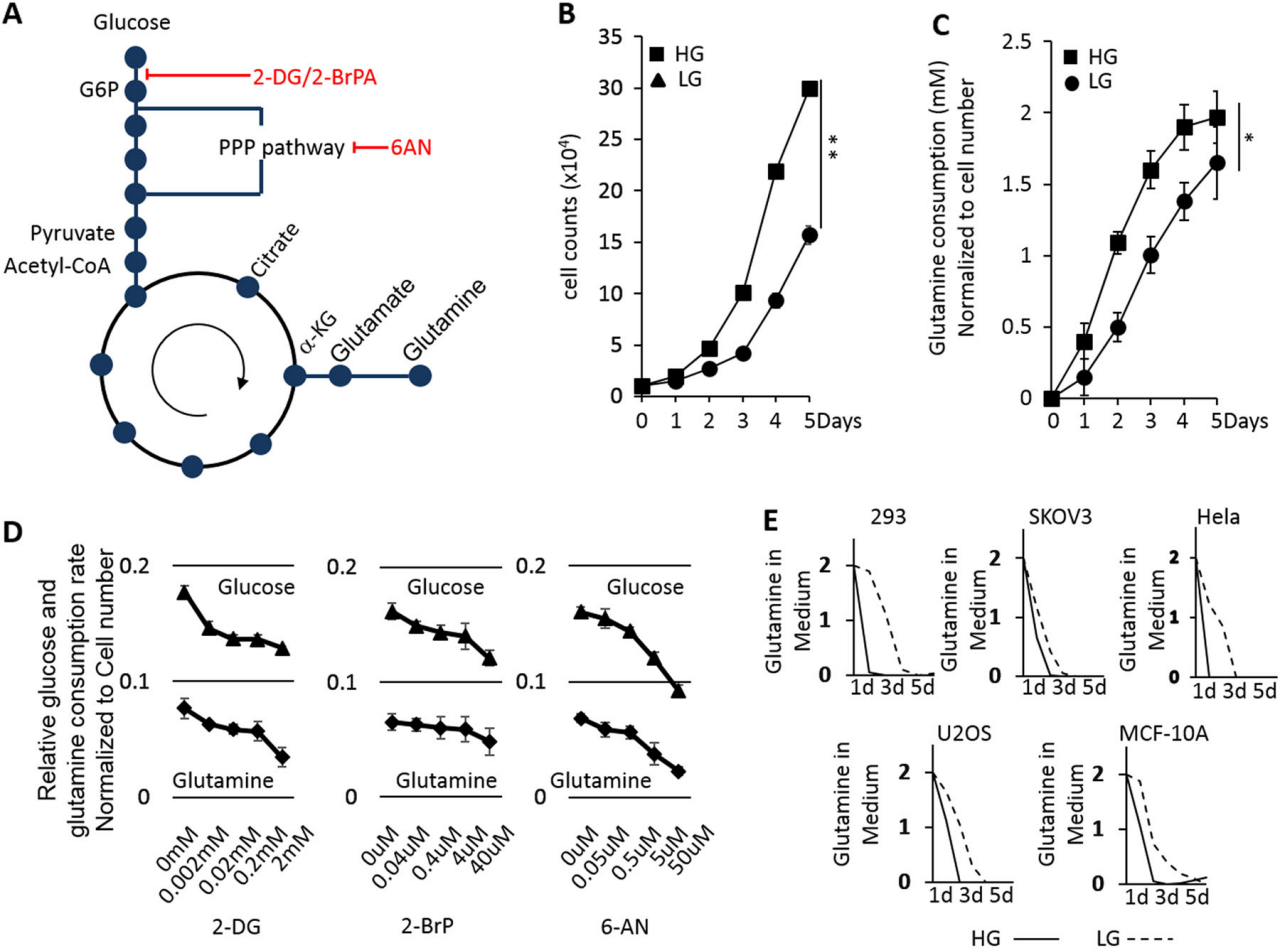
**a** The schematic illustration of glycolysis and glutaminolysis pathways interconnected by TCA cycle in proliferation cells. **b** Growth curve of MCF-7 cells in both high glucose (HG) and low glucose (LG) conditions. **c** Glutamine consumption measurement of MCF-7 cells under HG and LG culture conditions. **d** Glucose and glutamine consumption rate measurement in MCF-7 cells treated with glycolysis inhibitors 2-DG, 3-BP, and 6-AN with increased dosage. To ensure the comparability, the cell number for each condition are the same. **e** Glutamine consumption rate in different cell lines culturing in HG (solid line) or LG (dashed line) conditions. Initial glutamine is 2 mM. The error bars represent the SD of three independent replicates. *p < 0.05, **p < 0.01

Glucose and glutamine are two major carbon sources for cancer cells and both of them can enter the TCA cycle to produce energy (Fig. 1a). In order to investigate whether glycolysis impacts glutaminolysis, we cultured MCF-7 cells in high glucose (HG, 4.5 g/L glucose) medium and low glucose (LG, 1 g/L glucose) medium but supplied with the same initial amount of glutamine (2 mM). We did not observe obvious increased apoptosis associated with 1 g/L glucose culture condition for MCF-7 cells and MDA-MB-231 cells (data not shown). As expected, the cells cultured in HG medium showed a much faster proliferation than the cells in LG medium (Fig. 1b). To monitor the ability of glutamine consumption by each individual cell in HG and LG culture conditions, the glutamine consumption was normalized to cell number. Surprisingly, the cells cultured in LG medium exhibited retarded glutamine consumption as shown in Fig. 1c.

To simulate the LG condition, we also applied glycolysis inhibitors including 2-deoxyglucose (2-DG), 3-Bromopyruvate (3-BP), and 6-aminonicotinamide (6-AN) to restrict the glucose consumption^16^. As shown in Fig. 1d, MCF7 cells showed dose-dependent reduction of consumption of both glucose and glutamine at the individual cell level. As expected, the reduction of glutamine consumption in response to increased drug doses consistently followed the decreasing trend of glucose consumption, suggesting that the consumption of these two nutrients are correlated with each other in cancer cells. Such a trend was also observed in several other types of cells, including the non-transformed mammary gland epithelial cell MCF-10A and other types of cancer cells (Fig. 1e), indicating glycolysis activity impacts glutamine consumption without cell specificity.

### SIRT4-regulated glutaminolysis is influenced by glucose supply

**Fig. 2:**
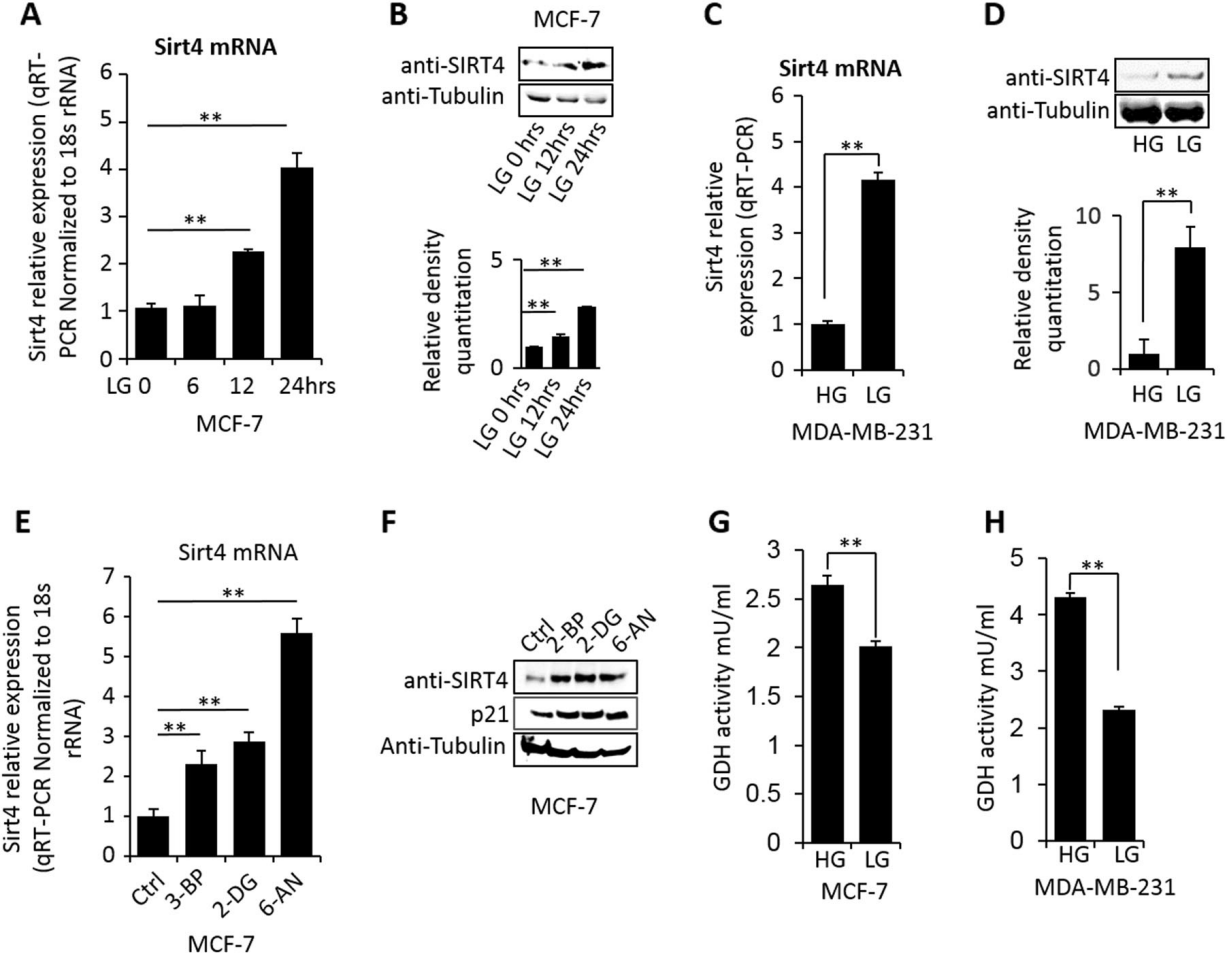
**a**, **b** *SIRT4* gene expression measured by mRNA or protein in MCF-7 cells culturing in LG condition. The densitometry analysis of western blot bands was shown. **c**, **d** *SIRT4* gene expression measured by mRNA or protein in MDA-MB-231 cells culturing in LG condition. The densitometry analysis of western blot bands was shown. **e**, **f** *SIRT4* gene expression and *p21* gene expression measured by mRNA or protein in MCF-7 cells treated by glycolysis inhibitors including 3-BP (40 uM), 2-DG (2 mM), and 6-AN(50 uM). **g**, **h** GDH activity assay in MCF-7 cells and MDA-MB-231 cells upon the cells were treated with LG condition. The error bars represent the SD of three independent replicates. *p < 0.05, **p < 0.01

SIRT4 is a known regulator of glutaminolysis in mitochondria via repressing GDH activity^14^. SIRT4 was demonstrated to be upregulated in response to DNA damage^13^. Thus, we wonder whether SIRT4 is responsible for the decreased glutamine consumption upon reduction of glucose consumption in cultured cancer cells. We selected MCF-7 and MDA-MB231 cells to detect the expression of *SIRT4* gene. When the cells were cultured in LG condition, the *SIRT4* expression showed a time-dependent increase (Fig. 2a,b). We also observed the upregulation of *SIRT4* by LG treatment in MDA-MB-231 cells at both mRNA and protein levels (Fig. 2c,d). LG condition can be mimicked by application of glycolysis inhibitors and *SIRT4* showed the corresponding increase in MCF-7 cells (Fig. 2e,f), suggesting that limiting glycolysis upregulates *SIRT4* expression. Correspondingly, *p21* was also upregulated upon glycolysis inhibitors treatment, indicating the halted cell growth (Fig. 2f), similar to the DNA damage effect. Importantly, when cells were cultured under LG condition, the GDH activity reduced significantly in MCF-7 and MDA-MB231 cells (Fig. 2g,h). Together with our previous result that CtBP promotes GDH activity^15^, we speculate a CtBP-SIRT4-GDH axis may coordinate glucose and glutamine metabolism.

### CtBP-repressed expression of SIRT4 is attenuated by LG treatment

**Fig. 3:**
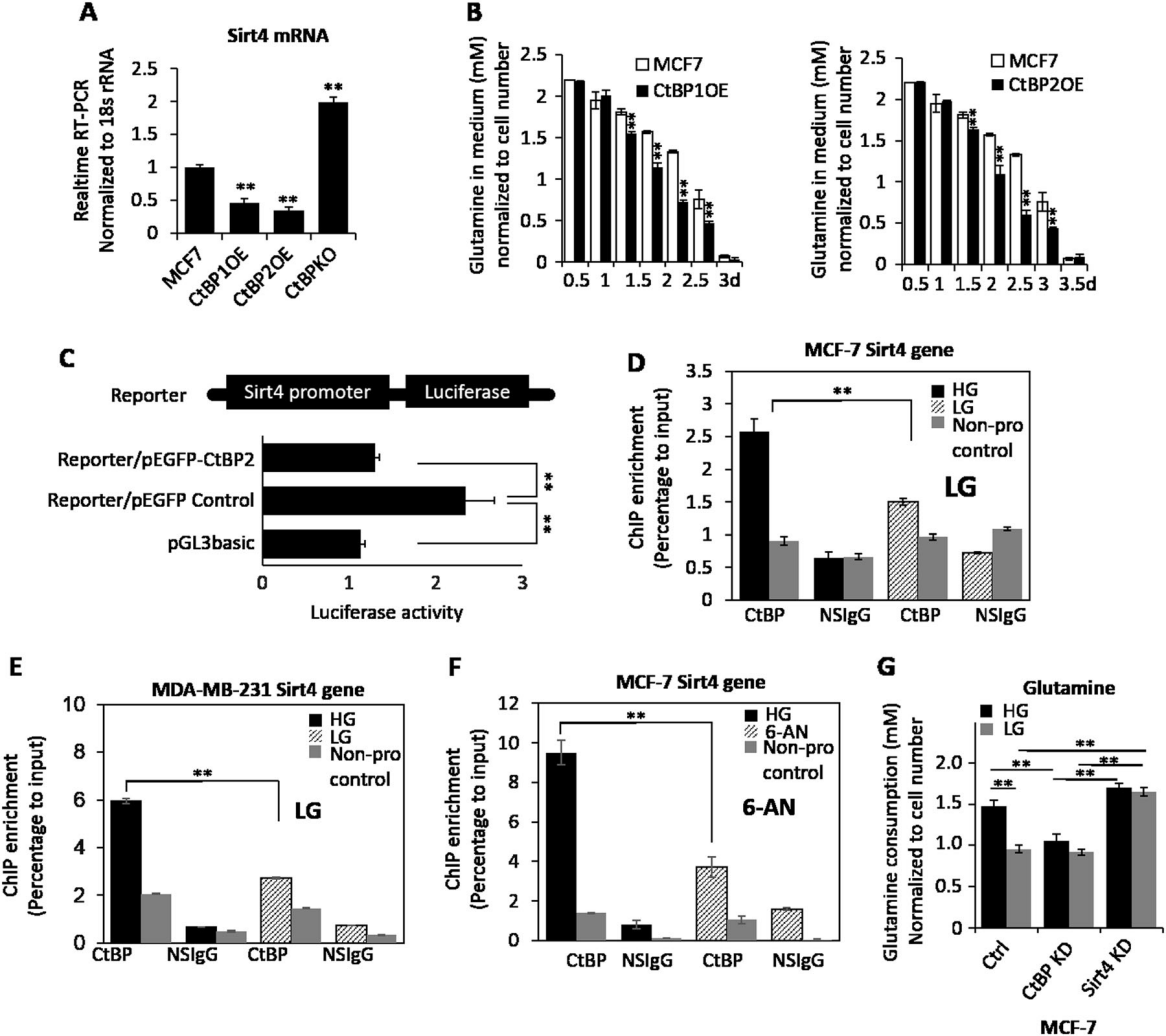
**a** *SIRT4* gene expression measured by mRNA when the MCF-7 cells were transfected with CtBP1 or CtBP2 overexpression vector, or the CtBP knockdown oligo. **b** Glutamine consumption measurement in MCF-7 cells with or without stable expression of CtBP1 (left) or CtBP2 (right). **c** *SIRT4* promoter activity assay using luciferase as a reporter, in response to CtBP2 co-transfection. **d**–**f** ChIP assay of CtBP binding at the *SIRT4* promoter in response to LG culture condition in MCF-7 cells (**d**), MDA-MB-231 cells (**e**), or 6-AN (50 μM) treatment in MCF-7 cells (**f**). A neighbor region of SIRT4 gene without transcripts (Non-pro) was used as a negative binding control region and nonspecific IGG (NSIgG) was used as negative control for chromatin pull down. The binding was shown as a percentage of input. **g** Glutamine consumption measurement in response to CtBP knockdown or *SIRT4* knockdown under HG and LG conditions. The error bars represent the SD of three independent replicates. *p < 0.05, **p < 0.01

We have identified CtBP as an important regulator of glutaminolysis pathway through repressing* SIRT4* gene expression^15^. To further investigate whether CtBP can directly repress *SIRT4* expression, we examined *SIRT4* expression in MCF-7 cells with stably overexpressed CtBP. The result demonstrated that *SIRT4* was repressed by overexpressed CtBP and knockdown of CtBP increased *SIRT4* expression significantly (Fig. 3a). As expected, these CtBP-overexpressing cells also showed a higher rate of glutamine consumption as indicated by decreased remaining glutamine in medium (Fig. 3b). Moreover, by cloning *SIRT4* promoter into the luciferase reporter vector, we observed that overexpression of CtBP significantly decreased luciferase activity of *SIRT4* promoter (Fig. 3c). Next, we investigated whether the repression effect of CtBP is also under the regulation of glucose level, we performed chromatin immunoprecipitation (ChIP) assay to assess CtBP binding at *SIRT4* promoter. Our results showed that LG culture reduced CtBP binding at *SIRT4* promoter significantly in MCF-7 and MDA-MB231 cells (Fig. 3d,e), suggesting there is a glucose metabolism-related regulatory role of CtBP in repressing* SIRT4* expression. Consistently, the glycolysis inhibitor 6-AN reduced CtBP binding at *SIRT4* promoter in MCF-7 cells (Fig. 3f). Next, we examined the glutamine consumption upon CtBP knockdown or *SIRT4* knockdown in both HG and LG conditions. CtBP knockdown decreased glutamine consumption only in HG condition, whereas* SIRT4* knockdown increased glutamine consumption in both HG and LG conditions (Fig. 3g), supporting the hypothesis that CtBP function requires the sufficient glucose supply, while *SIRT4* has a dominant role in regulating glutamine consumption.

### Dimerization of CtBP is regulated by glycolysis activity

**Fig. 4:**
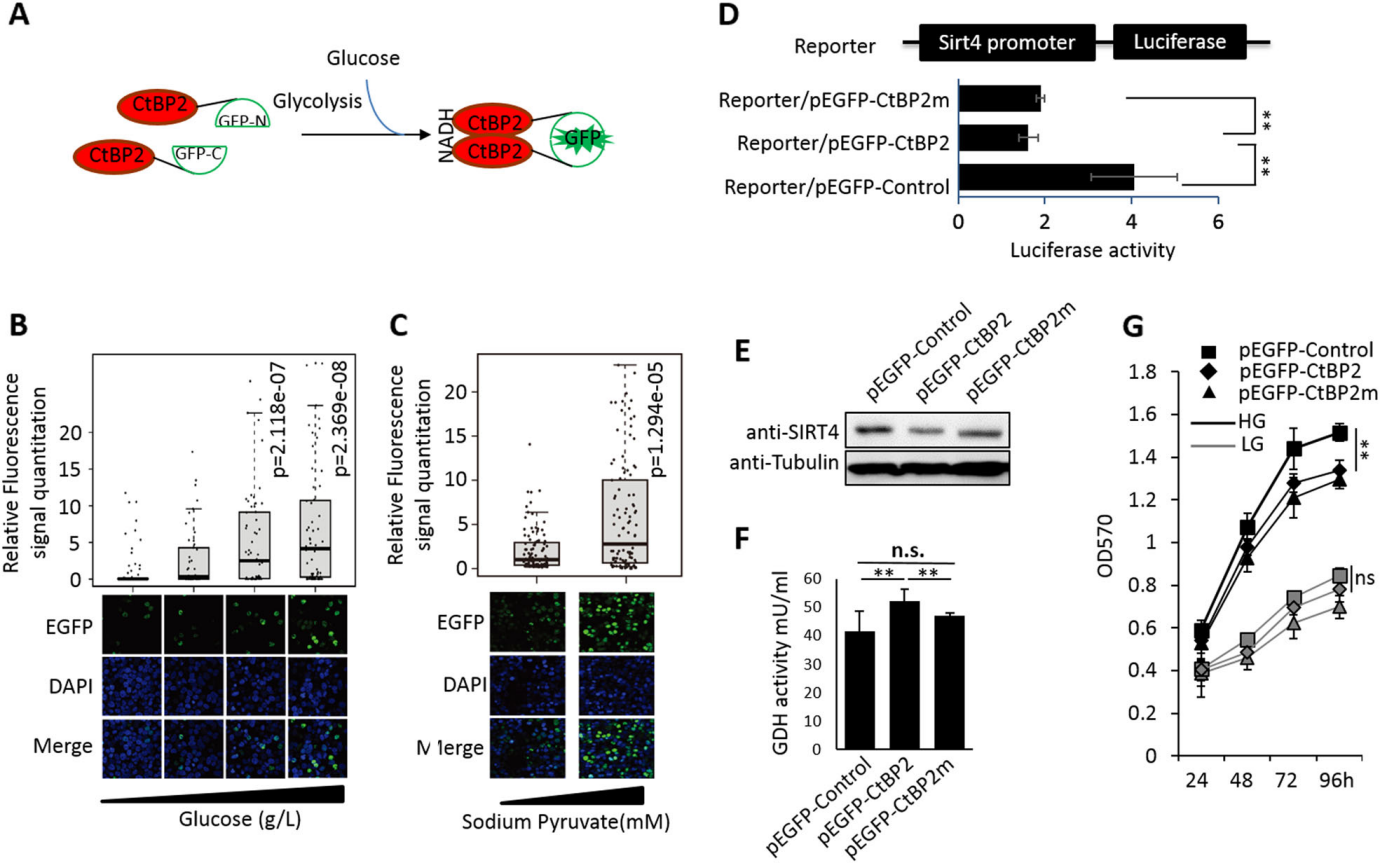
**a** Illustration of the application of BiFC technology to demonstrate CtBP dimerization. CtBP dimerization in response to **b** glucose or **c** pyruvate. Top is the box plot of signals from each bright fluorescent cell and bottom is the representative area with cells emitting BiFC fluorescent signal. **d** *SIRT4* promoter activity assay using luciferase as a reporter, in response to CtBP2 co-transfection or CtBP2 mutant (CtBP2m) co-transfection. **e** *SIRT4* expression in response to CtBP2 overexpression or CtBP2m overexpression determined by western blotting. **f** GDH activity in MCF-7 cells transfected with empty vector, CtBP2, or CtBP2m. **g** Cell growth measurement by MTT assay. The cells were transfected with empty vector, CtBP2, or CtBP2m and cultured in either HG or LG conditions. The error bars represent the SD of three independent replicates. *p < 0.05, **p < 0.01

As a well-studied co-repressor, dimerized CtBP is believed to have more potential to repress target gene expression. In order to demonstrate the dimerization status of CtBP in living cells, the bimolecular fluorescence complementation (BiFC) technology was employed. Transfection of both CtBP-GFP-N and CtBP-GFP-C constructs generates the intact green fluorescent protein (GFP) protein via CtBP dimerization to produce green fluorescence signal in the 293T cell nuclei (Fig. 4a). When the glucose supply in the culture medium was reduced, the formation of the GFP signal also decreased gradually, suggesting that glucose metabolic perturbation (i.e., glucose starvation) negatively regulates CtBP dimerization, which ultimately leads to the disruption of GFP signal (Fig. 4b). Similar to glucose, we also observed that pyruvate supplementation in medium increases GFP signal (Fig. 4c). To demonstrate that the dimerization of CtBP is critical in regulating SIRT4 expression, the SIRT4 promoter activity in response to wild-type CtBP or CtBP with dimerization defect^17^ was evaluated by luciferase reporter assay. As expected, SIRT4 promoter luciferase activity was strongly repressed by CtBP with intact dimerization activity but not by mutated CtBP (Fig. 4d). We further confirmed that the endogenous SIRT4 expression loses response to CtBP mutant but repressed by wild-type CtBP (Fig. 4e). Consistently, CtBP mutant only marginally increases GDH enzymatic activity (Fig. 4f). Finally, we observed that CtBP mutant loses the ability to promote cell proliferation in both HG- and LG-cultured cells. Taken together, these data further confirm that CtBP dimerization is under the regulation of cell metabolism status, presumably by NADH availability mainly from glucose metabolism, and *SIRT4* expression is subject to the regulation by CtBP dimerization status.

### MTOB inhibition of CtBP reverses glycolysis impact on glutamine consumption in cancer cells

**Fig. 5:**
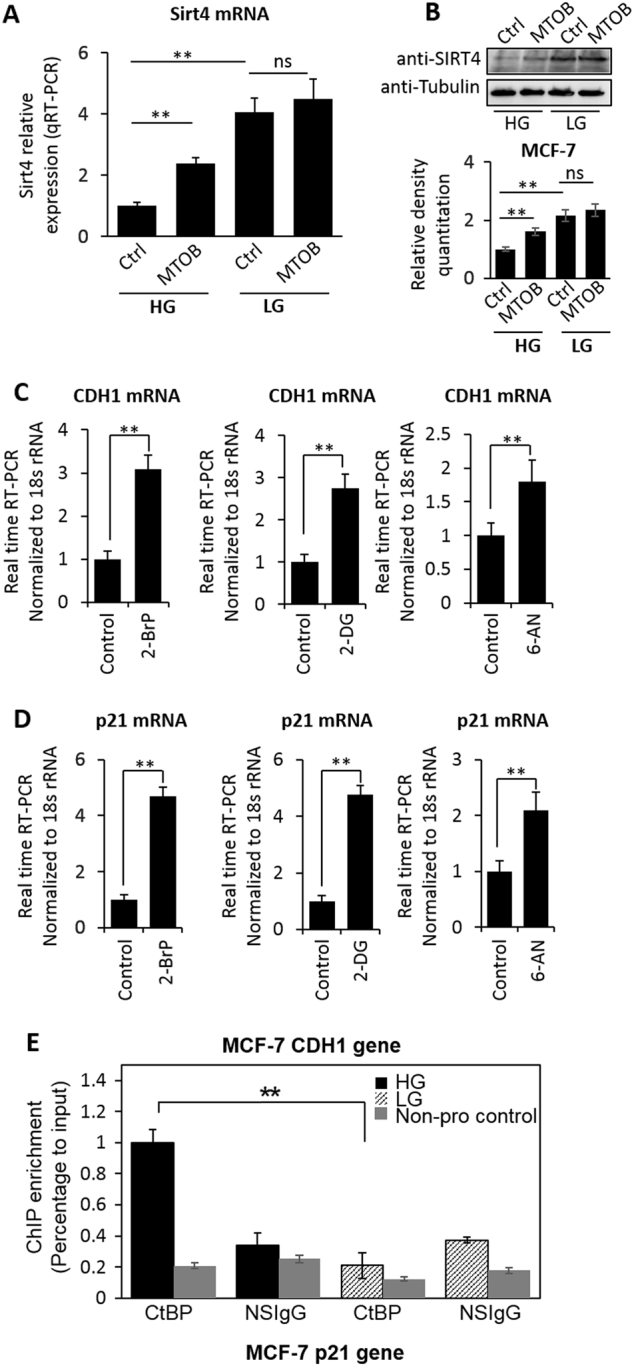
**a**,**b** *SIRT4* expression measurement by mRNA or protein in response to MTOB treatment under both HG and LG culture conditions. The densitometry analysis of western blotting bands was shown. **c**,**d** CDH1 and p21 mRNA measurement in MCF-7 cells in response to 3-BP, 2-DG, and 6-AN treatment. **e** ChIP assay of CtBP binding at *CDH1* gene promoter in response to LG culture condition in MCF-7 cells. A neighbor region of *SIRT4* gene without transcripts (Non-pro) was used as a negative binding control region and nonspecific IGG (NSIgG) was used as negative control for chromatin pulldown. The binding was shown as a percentage of input. The error bars represent the SD of three independent replicates. *p < 0.05, **p < 0.01

As CtBP regulates *SIRT4* expression via dynamically alternating its dimerization in response to cellular metabolic status, we investigated whether 4-methylthio-2-oxobutyric acid (MTOB) can directly inhibit CtBP and upregulate *SIRT4*, given that MTOB has been previously reported to inhibit the CtBP dimerization^18–20^. Consistent with the data that LG treatment abolished CtBP repression of *SIRT4*, MTOB stimulated expression of *SIRT4* only under HG condition but not under LG condition (Fig. 5a,b). These data suggest that LG condition and MTOB regulate *SIRT4* expression via the same mechanism, which is the alteration of CtBP dimerization. CtBP is also known to directly repress some other target genes such as *CDH1* (E-cadherin) and *p21*^21^. To assess whether CtBP repression of these target genes is also under influence of cell metabolism, the expression of both *CDH1* and *p21* genes were measured under the conditions of glycolysis inhibition by 3-BP, 2-DG, or 6-AN. As expected, both genes were upregulated significantly by these glycolysis inhibitors (Fig. 5c,d). Importantly, LG culture of MCF-7 cells significantly reduced the binding of CtBP to *CDH1* promoter as well (Fig. 5e), suggesting a change of cell metabolic activity directly interferes the CtBP-binding activity as co-repressor.

### CtBP mediates the glucose impact on cancer cell metabolic homeostasis

**Fig. 6:**
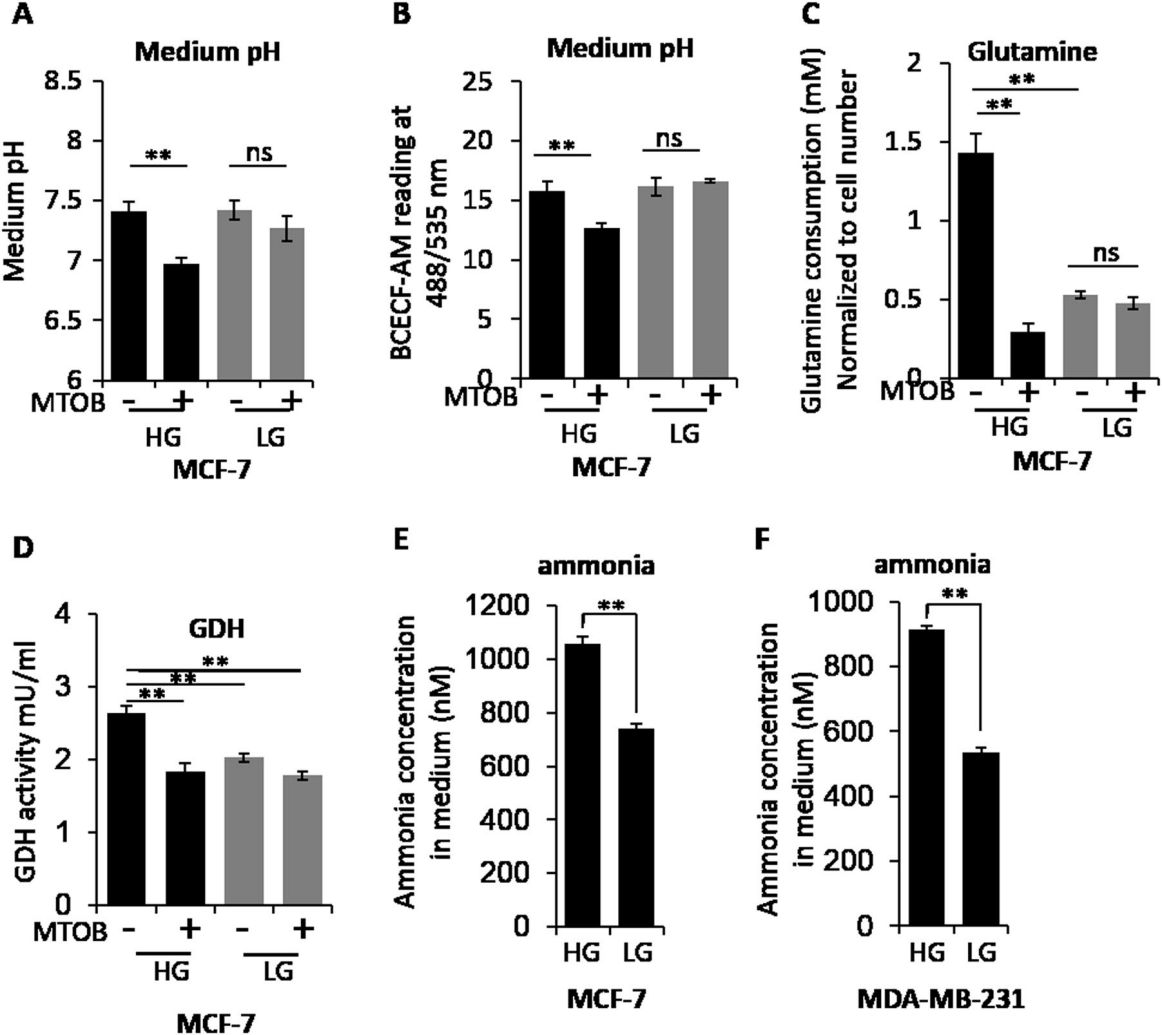
**a**,**b** Measurement of medium pH using pH meter or fluorescence probe when MCF-7 cells were cultured in HG and LG medium with or without the treatment by MTOB. **c** Glutamine consumption measurement in MCF-7 cells growing in HG and LG conditions. **d** GDH activity measurement in MCF-7 cells cultured in HG or LG conditions, with or without MTOB treatment. **e**,**f** Ammonia measurement in HG and LG medium culturing MCF-7 cells or MDA-MB231 cells. The error bars represent the SD of three independent replicates. *p < 0.05, **p < 0.01

Finally, we evaluated the impact of CtBP inhibition on cancer cell metabolic homeostasis. Consistent with our previous study^15^, MTOB only reduced the pH of MCF-7 cells significantly in HG condition but not in LG condition as indicated by the pH value and pH indicator (BCECA-AM; Fig. 6a,b). Furthermore, MTOB dramatically blocked the glutamine consumption in HG cultured MCF-7 cells but not in LG cultured cells (Fig. 6c). Consistently, the GDH activity was only significantly reduced by MTOB in HG-cultured MCF-7 cells (Fig. 6d). In addition, the LG-cultured cells showed lower initial GDH activity (Fig. 6d). We further measured the released ammonia in culture medium of both MCF-7 cells and MDA-MB-231 cells, and found LG-cultured cells secreted less ammonia into culture medium compared with HG-cultured cells (Fig. 6e,f), suggesting LG condition interrupts the glutamine consumption and the ammonia production in cancer cells. Collectively, our data indicate that there is a CtBP-SIRT4-GDH axis that coordinates the cell metabolic activity in taking glucose and glutamine as essential nutrients. The existence of this axis may explain the clinical relevance between glucose consumption and glutamine consumption by cancer cells.

## Discussion

The importance of amino acids, glucose, and fatty acids in cell proliferation has been investigated for a long time. However, the question that which one among these nutrients can be considered as the dominant one, in particular in the cancer scenario, is still unanswered^22–24^. Explanation of “Warburg effect” in a way to demonstrate the importance of glucose as a supplier of building block, beyond to be an energetic molecule, partially solves the puzzle^5,24^. However, some recent studies suggest glutamine, the most abundant amino acids in plasma, may contribute to cancer cell proliferation through the glutaminolysis pathway, which is eventually integrated into the TCA cycle^8,25^. There are a few studies that also proposed that it is the non-glutamine amino acid, especially the aspartate, acting as the critical rate-limiting element and having a critical role in cancer cell proliferation^23,26^. It is noteworthy that none of the above mentioned ingredients is able to fulfill all the needs of cell duplication. More or less, the other ingredients are required in different nutritional conditions. However, it may also be important to coordinate the metabolic pathways that process different substrates in order to achieve the largest output from raw nutrients as we reported in this study.

It is reasonable to speculate that there is a crosstalk between the glucose and glutamine metabolism in actively proliferating cells given that both glucose and glutamine are abundant and essential for tumor growth^27^. However, only very few studies attempted to illustrate the potential mechanisms and none of the previous research identified a universal mechanism applicable to proliferating cells^9,10^. The detailed mechanism that connects these two processes is unclear, partially owing to the complexity of metabolic pathways. In active proliferating cells, glucose is mainly consumed through anaerobic glycolysis pathway and a significant portion of carbon is converted to lactic acid that is transported out of cells. The serine synthetic pathway, or known as one-carbon cycle that stems from 3-p-Glycerol, and the PPP pathway, are all known to be upregulated in cancer cells and are essential to provide the building blocks for cell division. However, to meet the needs, the cells also need import glutamine as sources of both carbon and nitrogen. Glutamine is converted to α-ketoglutarate and integrated into the TCA cycle. Many recent observations indicate that most of the components of TCA cycle such as α-ketoglutarate, fumarate, and malate, etc. become more abundant and can be used as the primary synthetic materials. In particular, citrate is output to the cytosol for the synthesis of fatty acids. Therefore, our finding of CtBP-SIRT4-GDH regulatory pathway that mediates glycolysis impact on glutamine metabolism explains how the cancer cells coordinate these two critical processes to meet the need of their growth. In fact, previous studies observed that depletion of glucose caused cell growth inhibition and cell death in most of cell types even when glutamine supply is not suspended^28,29^. Thus, it seems that CtBP-SIRT4-GDH axis represents a mechanism for the cells to halt most of the metabolic activities, in order to preserve the opportunities for survival. Interestingly, shortage of glutamine also inhibits glucose uptake in pancreatic cancer cells^30^, suggesting these two essential ingredients for cell growth are both required for balanced metabolic activities and maintaining pH homeostasis may only be part of the balance^15^.

CtBP has several known enzymatic activities such as the NAD-dependent dehydrogenase activity and the lysophosphatidic acetyl-CoA transferase activity^31,32^. However, none of these enzymatic activity is known to be essential for CtBP function, in particular, as transcriptional co-repressor in the nucleus. In comparison with other well-known co-repressors such as RIP140, NCOR1, NURD, etc., CtBP has rarely been reported to be an important regulator of cell metabolism, including in a disease context, except our recent discovery that CtBP represses SIRT4 gene expression and regulates intracellular pH homeostasis via modulating glutaminolysis^15^. However, CtBP has been known as a redox sensor for many years, which inevitably promotes us to speculate that CtBP must have an unknown function in mediating the metabolic signal to fine-tune the cell activities, including the metabolic activities. Not surprisingly, there are many metabolic pathway regulators identified to be targets of CtBP transcriptional regulatory function including SIRT4 in the genome-wide profiling of CtBP targets in cancer cells^19^.

Dimerization of CtBP is critical for its repressive function. However, previous studies fail to demonstrate the dynamics of the dimerization activity in living cells^33^. In our study, we applied a technology known as BiFC to directly demonstrate the existence of CtBP dimer^34^. To our knowledge, this is the first time that CtBP dimerization can be directly viewed in living cells. Moreover, the formation of CtBP dimer is subjected to the regulation of cell metabolism status. By culturing cells in various level of glucose, CtBP dimerization shows dynamic change revealed by GFP signal. The ability to observe the dimerized CtBP and the confirmation of the accordance between CtBP dimerization and cell metabolism paves the way to study how CtBP mediates the cell metabolism response in regulating gene expression profile. In fact, besides *SIRT4* gene, we also observed that CtBP is a mediator of cell metabolic signal in regulating its previous identified targets such as the well-known tumor suppressors *E-cadherin* and *p21*, suggesting the mechanism we report here may represent a general mechanism that dictate the nuclear transcriptional activity by cell metabolic status.

## Material and Methods

### Cell culture

MCF-7 cells and MDA-MB-231 cells were maintained in regular Dulbecco's modified Eagle’s medium supplemented with 10% (v/v) fetal bovine serum and penicillin–streptomycin (Invitrogen). For knockdown experiments, the procedure and oligo for CtBP knockdown are the same as described before^18^. SIRT4 knockdown oligos were purchased from Santa Cruz as mixed oligo pool and the procedure followed the manual provided by the manufacturer. For overexpression experiments, CtBP expression vector was transfected into the indicated cells as described^19^.

### Chemicals and reagents

MTOB, 3-Bromopyruvate (3-BP), and 6-AN were purchased from Sigma-Aldrich. MTOB was dissolved in medium to 250 mM and diluted to 10 mM final concentration in cell culture. The antibody of CtBP used for ChIP was purchased from Santa Cruz Biotechnology and is cross-reactive with both CtBP1 and CtBP2. The glutamine, glucose, and ammonia colorimetric assay kits were purchased from Biovision (USA) and Bioassay Systems (USA).

### Reverse transcriptase-PCR and western blotting

Both experiments were performed following the standard protocol as previously described^15^.

### Chromatin immunoprecipitation

All ChIP experiments were carried out as described^19^.

### pH measurement

For culture medium, the medium was removed from the culture dish immediately after the dishes leaving the incubator and the pH was measured using regular lab pH meter. For measurement of cytoplasmic pH, a fluorescence probe BCECF-AM was applied. The fluorescence signal of BCECF-AM is positively correlated with intracellular pH and negatively correlated with intracellular acidity. BCECF-AM stock (5 mM) was purchased and 10 μM final concentration was applied to the cells. The cellswere incubated with BCECF-AM for 30 min. The extra BCECF-AM solution from the cells were washed away with phosphate-buffered saline (PBS) for three times. Next, the cells were kept in PBS and the signals read using the fluorescent plate reader with excitation at 480 nm and emission at 535 nm.

### Measurements of glutamine, glucose, and ammonia in the medium

The measurements of these metabolites were performed according to the manual provided by the manufacturer of these colorimetric kits. Briefly, the cell culture medium was collected and centrifuged to remove the debris. The medium then was diluted for 2–10 times depending on the applications. The substrates and the enzymes were added to each sample, and were allowed to react for a required time secured from light. Then the colorimetric signals were obtained by reading the plates at assigned wavelength. The detailed manuals are provided by the manufacturer (Bioassay Systems). For ammonia measurement, the readings include both ammonia and ammonium. The procedure in analyzing GDH activity followed the manual provided by the manufacturer of GDH activity assay kit (Biovision).

The measurements were also performed when sufficient medium was available, using the Bioprofile Flex analyzer (Nova Biomedical). Briefly, 800 μL of the culture medium were aspirated into the tray module of the Bioprofile Flex analyzer (Nova Biomedical) to measure glucose consumption and glutamine consumption simultaneously. Data were normalized to the cell number or protein concentration in each culture well.

### Luciferase reporter assay

The MCF7 cells were transfected with pGL3-basic-SIRT4p together with empty vector (pEGFP) or CtBP expression vector (pEGFP-CtBP2/pEGFP-CtBP2m). After 16 h of transfection, the cells were then washed twice with PBS and lysed for 15 min. The lysates were collected by centrifugation at 13,000 r.p.m. and 20 μl of lysate was used to measure the luciferase reporter activity. The luciferase activity was normalized to *Renilla* luciferase activity from the co-transfected internal control plasmid pRL-TK. A dual-luciferase assay was performed using the Dual-Luciferase Reporter Assay System (Promega) on the Pekin Elmer plate reader with the auto-injection system.

### Bimolecular fluorescence complementation

The backbone of BiFC plasmids was obtained from Addgene (pBiFC-VC155/22011 and pBiFC-VN155(I152L)/27097)^35^. CtBP coding sequence was cloned into these two vectors according to the available restrictive sites.

### Statistical analysis

All the error bars represent the SD of the mean from at least three independent biological replicates, unless otherwise indicated. Comparisons between two groups were done using unpaired Student’s *t*-test. *P* < 0.05 was considered statistically significant.
